# Supplementary material for: How attitudes of state and community leaders regarding health equity and social determinants of health are associated with behavioral intentions to improve population health
Source: PLoS One. 2024 Apr 18;19(4):e0301450. doi: 10.1371/journal.pone.0301450 (PMC11025853; doi:10.1371/journal.pone.0301450)
Supplement: S1 Checklist — (PDF) [file pone.0301450.s001.pdf]

STROBE Statement—checklist of items that should be included in reports of observational studies

	Item No.	Recommendation	Page No.	Relevant text from manuscript
**Title and abstract**	1	(*a*) Indicate the study's design with a commonly used term in the title or the abstract	2	We designed a survey instrument…and fielded the survey to 18,367 state and community leaders.
		(*b*) Provide in the abstract an informative and balanced summary of what was done and what was found	2	Survey respondents viewed social determinants of health and health equity as important factors influencing population health. Additionally, two attitudes were most associated with leaders' intentions to promote population health—"self-efficacy"…and "behaviors valued by important others".
**Introduction**				
Background/rationale	2	Explain the scientific background and rationale for the investigation being reported	3-4	Despite the potential role of leaders in influencing health and well-being, little is known about their attitudes regarding their organization's perspective on what shapes health, or how their attitudes translate into actions that improve population health. There is a need to fill this gap in the evidence base…. To our knowledge, no other surveys have sampled a nationally representative group of diverse, cross-sectoral leaders and asked about their attitudes toward health and well-being.
Objectives	3	State specific objectives, including any prespecified hypotheses	4	The objectives of this study are twofold: (1) to understand leaders' attitudes with regard to their organization's perspective on population health and (2) to

				identify the attitudes associated with leaders' perspectives regarding their organization's intention to engage in behaviors that advance population health.
**Methods**				
Study design	4	Present key elements of study design early in the paper	5	We created a conceptual framework to inform the development of the…survey instrument and sample plan.
Setting	5	Describe the setting, locations, and relevant dates, including periods of recruitment, exposure, follow-up, and data collection	7	We fielded the survey in two phases—Phase 1 ran from October 9, 2020, to May 31, 2021…and Phase 2 ran from September 28, 2021, to March 31, 2021…. Each survey phase was fielded over a 22-week period.
Participants	6	(*a*) *Cohort study*—Give the eligibility criteria, and the sources and methods of selection of participants. Describe methods of follow-up<br>*Case-control study*—Give the eligibility criteria, and the sources and methods of case ascertainment and control selection. Give the rationale for the choice of cases and controls<br>*Cross-sectional study*—Give the eligibility criteria, and the sources and methods of selection of participants	7	The target population was organizational leaders in city and state government and leaders of for-profit and nonprofit organizations…. We drew one sample from state-level organizations or agencies in all 50 states, and we drew the other sample from city-level organizations and agencies in 325 cities across the U.S. Both samples consisted of leaders in a wide array of sectors.
		(*b*) *Cohort study*—For matched studies, give matching criteria and number of exposed and unexposed<br>*Case-control study*—For matched studies, give matching criteria and the number of controls per case		N/A (cross-sectional study)
Variables	7	Clearly define all outcomes, exposures, predictors, potential confounders, and effect modifiers. Give diagnostic criteria, if applicable	9-11	Table 3. Question language for framework constructs
Data sources/ measurement	8*	For each variable of interest, give sources of data and details of methods of assessment (measurement). Describe comparability of assessment methods if there is more than one group	9	We calculated scores for each framework construct based on responses to the survey questions for that construct; we

				did this by calculating the mean for the grid list items for that question. Scores range from 1 to 5, with higher scores indicating more positive attitudes.
Bias	9	Describe any efforts to address potential sources of bias	8	We conducted our analyses…to account for the complex sample design and to apply the weighting for selection probability with nonresponse adjustments for the race and gender of the respondents and for the region, city population size, and city area deprivation index scale, and normalized to roughly equate each stratum.
Study size	10	Explain how the study size was arrived at	8	We fielded the survey to 18,367 leaders and received survey responses from 5,450 leaders, achieving a response rate of 32 percent.

Quantitative variables	11	Explain how quantitative variables were handled in the analyses. If applicable, describe which groupings were chosen and why		See #7 and #8, above.
Statistical methods	12	(*a*) Describe all statistical methods, including those used to control for confounding	14	We conducted a regression analysis to assess which attitudes predicted behavioral intention. For this analysis, we selected the backward elimination procedure, specifically because it is well-suited to exploratory research…. However, the backward elimination procedure is vulnerable to multicollinearity, resulting in unstable regression results. Therefore, we conducted checks to assess multicollinearity and stability of the results.
		(*b*) Describe any methods used to examine subgroups and interactions		N/A (we did not examine subgroups or interactions)
		(*c*) Explain how missing data were addressed	16	We … [used] backward stepwise elimination, with the criterion for

				inclusion set at 0.05, listwise deletion of missing data, and two-tailed tests of statistical significance.
		(*d*) *Cohort study*—If applicable, explain how loss to follow-up was addressed<br>*Case-control study*—If applicable, explain how matching of cases and controls was addressed<br>*Cross-sectional study*—If applicable, describe analytical methods taking account of sampling strategy	8	We conducted our analyses…to account for the complex sample design and to apply the weighting for selection probability with nonresponse adjustments for the race and gender of the respondents and for the region, city population size, and city area deprivation index scale, and normalized to roughly equate each stratum.
		(*e*) Describe any sensitivity analyses		N/A (we did not conduct any sensitivity analyses)
**Results**				
Participants	13*	(a) Report numbers of individuals at each stage of study—eg numbers potentially eligible, examined for eligibility, confirmed eligible, included in the study, completing follow-up, and analysed	8	We fielded the survey to 18,367 leaders and received survey responses from 5,450 leaders, achieving a response rate of 32 percent.
		(b) Give reasons for non-participation at each stage		N/A (no stages in our study)
		(c) Consider use of a flow diagram		N/A (no stages in our study)
Descriptive data	14*	(a) Give characteristics of study participants (eg demographic, clinical, social) and information on exposures and potential confounders	8	Respondents primarily identified as male (57 percent); white and non-Hispanic (76 percent); liberal (50 percent), as opposed to conservative, moderate, or libertarian; working five or more years in their current job (58 percent); and 50–69 years of age (62 percent).
		(b) Indicate number of participants with missing data for each variable of interest		N/A (we used listwise deletion)
		(c) *Cohort study*—Summarise follow-up time (eg, average and total amount)		N/A (cross-sectional study)
Outcome data	15*	*Cohort study*—Report numbers of outcome events or summary measures over time		N/A (cross-sectional study)
		Case-control study—Report numbers in each exposure category, or summary measures of exposure		N/A (cross-sectional study)
		Cross-sectional study—Report numbers of outcome events or summary measures	13-14	Table 4. Comparisons among framework constructs
Main results	16	(*a*) Give unadjusted estimates and, if applicable, confounder-adjusted estimates and their precision	13-14; 16-17	Table 4. Comparisons among

		(eg, 95% confidence interval). Make clear which confounders were adjusted for and why they were included		framework constructs<br>Table 5. Standardized coefficients for framework constructs associated with behavioral intention
		(*b*) Report category boundaries when continuous variables were categorized		N/A (variables not categorized)
		(*c*) If relevant, consider translating estimates of relative risk into absolute risk for a meaningful time period		N/A (no risk estimates)
Other analyses	17	Report other analyses done—eg analyses of subgroups and interactions, and sensitivity analyses		N/A (no other analyses done)
**Discussion**				
Key results	18	Summarise key results with reference to study objectives	22	Leaders reported positive attitudes related to their organizations engaging meaningfully with residents, collaborating across sectors, addressing inequities in the health sector, promoting equitable resource allocation, and investing in programs that prioritize the well-being of disadvantaged communities, children, and families. At the same time, leaders cited various barriers that inhibit their organization's ability to take these actions…. The strongest predictor of behavioral intention is the leader's confidence in their organization's ability to take action ….to improve population health. The second strongest predictor was the leader's belief regarding how much certain groups…want the organization to take action.
Limitations	19	Discuss limitations of the study, taking into account sources of potential bias or imprecision. Discuss both direction and magnitude of any potential bias	25	We believe this is the first survey that has documented the health attitudes of a nationally representative sample of leaders.
Interpretation	20	Give a cautious overall interpretation of results considering objectives, limitations, multiplicity of analyses, results from similar studies, and other relevant evidence		See #18, above.
Generalisability	21	Discuss the generalisability (external validity) of the study results		N/A (specific study population, not intended to be generalizable)

**Other information**			
Funding	22	Give the source of funding and the role of the funders for the present study and, if applicable, for the original study on which the present article is based	N/A (not provided in manuscript per journal submission instructions)

*Give information separately for cases and controls in case-control studies and, if applicable, for exposed and unexposed groups in cohort and cross-sectional studies.

Note: An Explanation and Elaboration article discusses each checklist item and gives methodological background and published examples of transparent reporting. The STROBE checklist is best used in conjunction with this article (freely available on the Web sites of PLoS Medicine at http://www.plosmedicine.org/, Annals of Internal Medicine at http://www.annals.org/, and Epidemiology at http://www.epidem.com/). Information on the STROBE Initiative is available at www.strobe-statement.org.
